# Glycerite of Kephaline

**Published:** 1877-10

**Authors:** 


Glycerite of Kephaline.—There is a strong tendency to seek remedial agents in animal organisms, and the favor those already produced have received from the profession, indicates that good results have followed the use of them for the cure of disease. Pepsin is now so generally employed, that it may be regarded as an acceptable agent, whose efficiency is above question, and which is placed in the time-honored list. No physician would think of combatting aberrations of the digestive functions without seeking its good offices.

Isolated pancreatic juice has more recently sought professional endorsement. To Dobell the credit belongs of having first presented it to the profession. Its value is not so well determined as that of pepsin, and yet there is but little doubt that it is an excellent addition to our list of remedial agents. Admitting, as we must, that pancreatic juice is decomposed in the stomach by the acids, nevertheless it does not necessarily invalidate its use, because it will emulsify the fat and oils before they are taken into the stomach, and in consideration of the facts that the fats and oils previously emulsified pass through the stomach in a condition ready for appropriation, pancreatine, by previously emulsifying these, may prove exceedingly valuable in certain morbid conditions of the system. Ptyaline has been introduced as a chemical to supply the place of the normal saliva, but of its value I am not prepared to speak.

The last and most important attempt to appropriate the products of organic chemistry to the purposes of therapeutical science, is the isolation of protagon from the brains of animals, and wheat and its administration in diseases characterized by general vital deterioration.

Chemical research by Hentier and others, has demonstrated a decided deficiency of protagon in those whose intellectual powers decay, whether prematurely or by the natural changes incident to advanced life. The same deficiency is characteristic in those who destroy their vital powers by natural or unnatural sexual excesses; as also in those who overtax their intellectual powers by brain work. While histological research has demonstrated the relation between brain deterioration and deficiency of protagon, Routh, of London, Tilbury Fox, Tempini, of Italy, and Professor Hammond, of New York, give corroborative testimony that fish, oysters, eggs and other rich foods in organic phosphorous compounds, are efficient agents in the restoration of the absent amount of protagon.

Dr. Charles G. Polk, of Philadelphia, who has spent much time in chemico-histological research, and whose investigations of those dead of tubercular disease seems to determine a direct relation between deficiency of protagon, and the development of tubercular phthisis, says in the August 2d, 1873, number of the *Medical and Surgical Reporter*, that in 1857, while engaged in the chemico-histological study of consumption, that he found that there is always a deficiency of the phosphorized brain principles in the medulla oblongata, cerebellum, and at the base of the cerebrum, and that this deficiency is very strongly evinced in the nerve mass near the origin of the eighth pair of nerves.

Dr. Polk having determined the deficiency of protagon in blood and nerve masses as the characteristic feature of phthisis, conceived the idea that the remedy for the disease was the restoration of the missing phosphorous compounds to the system. Upon analysis of the brain, he determined that the protagon or morphological brain principle is hypophosphorous acid of the tribasic variety, in union with the alkaline salts, glycerine, olein and albumen.

In accordance with this determination, he formed these isolated brain principles into a glycerite, and employed it in diseases depending upon general vital deterioration. The results have been universally successful. In incipient phthisis the isolated brain principles have given results far more successful than heretofore accrued from the use of cod liver oil. I have had numerous opportunities of witnessing the employment of both Dr. Polk's protagon and glycerite of kephaline since 1869, and I can recall many cases in which there were very strong evidences of tubercular disease, but which rapidly improved under the use of the glycerite of kephaline. One case, especially, Mr. S., a member of a well known firm of surgical instrument manufacturers. In October, 1869, when he came under Dr. Polk's treatment, he was an apparantly hopeless case of phthisis. He was placed on glycerite of kephaline, and in six months was apparently in the enjoyment of good health. Since then, I have been a witness to equally satisfactory results in several dozen cases, and there is no doubt in my mind but that it is a very important contribution to the therapeutics of this malady.

To claim that it is a specific for tubercular consumption, would be claiming too much. It is not entitled to such a reputation, and the attempt to exalt it to this position will prove unsuccessful. Its value consists in restoring back to the system the alkaline hypophosphites which are deficient in the blood and nerve masses, and thus restoring the integrity of the functions to which these are so indispensable, namely, the generation of the nerve force transmitted for digestion, assimilation, nutrition and respiration through the eighth pair of nerves.

Without venturing into an extended analysis of the pathology and tiology of tubercular diseases, there are a few points right here suggested, to which it may not be irrelevant to allude. The lesions which characterize phthisis are mainly lesions of these very functions. Pancreatic deficiency, which Dobell supposes to be the developing cause of the disease, the primary lesion from which the other lesions accrue, is but an expression of impaired function of the excito-nutrient nerve system. Duodenal dyspepsia, so accurately described by James Clark, and which he declared to be the earliest symptom of tubercular trouble, is, in a large measure, a consequence of pancreatic deficiency, and depends upon the acid condition of the small intestines, which Bennett regarded as the immediate agent in the production of most of the lesions essentially connected with the malady, as the more than neutralization of the normally alkaline pancreatic juice, and consequent interference with the emulsification of the oleaginous constituents of the food, whereby an excess of the albuminous portion enters the blood, while the carbonaceous portion is, in a great measure, lost by not being prepared for assimilation.

In consequence of deficient emulsification of the fatty elements, butyric fermentation takes place, and the acid condition so graphically described by Bennett, is produced. It thus appears, and sanctioned by high authority, that tubercular disease, or rather the development of the tubercle, is the immediate consequence of an anterior cause. The anterior cause must be sought in the nerve masses, from which the abdominal viscera derives its nerve influence. Thus we are brought to the pneumogastric, the medulla oblongata, and the cerebellum. It is in these Dr. Polk found that the hypophosphites are uniformly deficient. If the great determining cause be in a deficiency of the hypophosphites in these nerve masses, the rational indication would be their restoration.

Churchill, of Paris, who is, I believe, justly regarded as the author of the phosphorous theory of consumption, as early as 1857 contended that the disease originated in a deficiency of phosphorous in the system, in an oxidizable form, but he did not make any chemical analysis to determine the location or formula of the missing element; consequently, we must infer that he arrived at his conclusions entirely on theoretical grounds, while Dr. Polk based his conclusions on actual chemical analysis of the blood and brain of those dead with phthisis.

Dr. Churchill introduced into medical practice laboratory-made hypophosphites of calcium and sodium, while Dr. Polk first isolated and employed brain hypophosphites in the treatment of tubercular consumption, and the success he has had with his glycerite of kephaline and Lœflund's extract of malt, indicates a decided superiority of this treatment over that usually employed by physicians.

In other cachectic and devitalized conditions of the system, the results with these agents exceed those derived from the usual tonics and alteratives. In nerve and brain exhaustion, glycerite of kephaline is without a rival—no agent compares with it. In impotency it is almost specific, in the absence of organic defect. The dose is about fifteen to twenty drops, given with malt vegetable tonics or in water, three times a day. The following is the formula:

℞. Isolated cerebrate or hypophosph. of ammonium . 8 parts.
Isolated cerebrate or hypophosph. of potassium. . 3 parts.
Isolated cerebrate or hypophosph. of calcium. . . . 8 parts.
Isolated cerebrate or hypophosph. of magnesium. 3 parts.

Isolated cerebrate or hypophosph. of sodium....8 parts.
Glycero hypophosphorous acid (isolated).......5 parts.
Cerebric acid.............................5 parts.
Pure glycerine............................60 parts.

The hypophosphites are isolated almost chemically pure by macerating protagon in bisulphide of carbon, and precipitating at a low temperature. The hypophosphites thus obtained are very soluble in the mixture of the glycerine and the acids. The preparation is almost as clear as the glycerine, and has an agreeable acid taste. At present, it is only manufactured by A. C. Dung, of New York city. The writer is in no manner concerned in its production or sale, and consequently cannot answer individual inquiries.—*Detroit Medical Journal.*

